# The circuitry of sex

**DOI:** 10.7554/eLife.22215

**Published:** 2016-11-15

**Authors:** Joel Levine

**Affiliations:** Department of Biology, University of Toronto Mississauga, Mississauga, Canadajoel.levine@utoronto.ca

**Keywords:** sexual behaviour, sexual-dimorphism, copulation, doublesex, *D. melanogaster*

## Abstract

Neural circuits that control copulation in male flies have been identified.

**Related research article** Pavlou HJ, Lin AC, Neville MC, Nojima T, Diao F, Chen BE, White BH, Goodwin SF. 2016. Neural circuitry coordinating male copulation. *eLife* **5**:e20713. doi: 10.7554/eLife.20713

Reproduction is widely considered to be the *raison d’etre* of life on Earth, and sex in the animal kingdom can take many forms. Some organisms – including worms and snails – are hermaphroditic and can self-fertilise to produce offspring (Leonard, 2006; Thomas et al., 2012). In other, perhaps more familiar, species, males and females mate to produce the next generation.

Given the importance of reproduction, it is not surprising that animals can devote much of their adult lives to, and employ all of their senses in, reproductive behaviours – whether that is seeking out mates and courting, or assessing the environment to see if it is a good time to reproduce. Moreover, cognitive processes such as memory, learning and decision-making are also harnessed to optimize reproductive success (Reif et al., 2002).

The vinegar fly *Drosophila melanogaster* is a model organism that shares its history and habitat with people (Keller, 2007). The mating habits of the fly have been closely observed in both the wild and the laboratory (Markow, 2015; Villella and Hall, 2008). What we know is: a male vinegar fly will court a female and, if she so chooses, they will mate. The male first uses his genital claspers to couple his genitals to those of the female. Next, the penis and associated branches emerge from his genital region and are inserted into her vaginal area as they copulate (Kamimura, 2010). Sex between vinegar flies typically lasts about 20 minutes, with the transfer of sperm and ejaculate being completed after about 9 minutes (Gilchrist and Partridge, 2000). When mating is finished, the male withdraws, and the claspers release.

The male’s penis is controlled by the coordinated interaction of about ten muscles in his genital region (Kamimura, 2010). However, until recently, relatively little was known about the way these muscles were controlled by the nervous system. Now, in eLife, Stephen Goodwin, Hania Pavlou and co-workers, at the University of Oxford, the National Institute of Mental Health and McGill University, report that they have identified the neural circuits that coordinate copulation in male vinegar flies (Pavlou et al., 2016).

Based on previous findings, Pavlou et al. knew that the neurons controlling copulation in male flies would be located in the fly’s nerve cord, which is equivalent to the spinal cord in humans (Hall, 1979; Ferveur and Greenspan, 1998). They looked for a population of neurons that expressed both a gene called *doublesex* and a neurotransmitter called glutamate. They did this because *doublesex* is an important gene that generates differences in the anatomy and behaviour of males and females, and because glutamate is associated with the motor neurons that instruct muscles to move (Rideout et al., 2010; Daniels et al., 2008). Also, *doublesex*-positive neurons were already known to control, amongst other things, the courtship songs of male vinegar flies (Rideout et al., 2007; Rideout et al., 2010; Shirangi et al., 2016).

About 80 such neurons were found in the male flies. Further experiments showed that when these neurons were triggered prior to copulation, the males would court but not copulate. On the other hand, when these neurons were triggered during copulation, the males would not terminate the act and separate. Based on these findings, Pavlou et al. suggested that these neurons control the muscles that contract or relax to move the penis during copulation. They also identified a set of interneurons that suppress these 80 or so motor neurons. These interneurons expressed both *doublesex* and the neurotransmitter called GABA.

The motor neurons and interneurons form the wires of a simple circuit that regulates the muscles (Figure 1). But one more type of neuron was required to complete the circuit: an input. Pavlou et al. found these missing connections in the form of sensory neurons linked to hair-like bristles positioned near the penis. These neurons express *doublesex* and the neurotransmitter called acetylcholine, and they project to the interneurons, motor neurons and brain.

**Figure 1.**
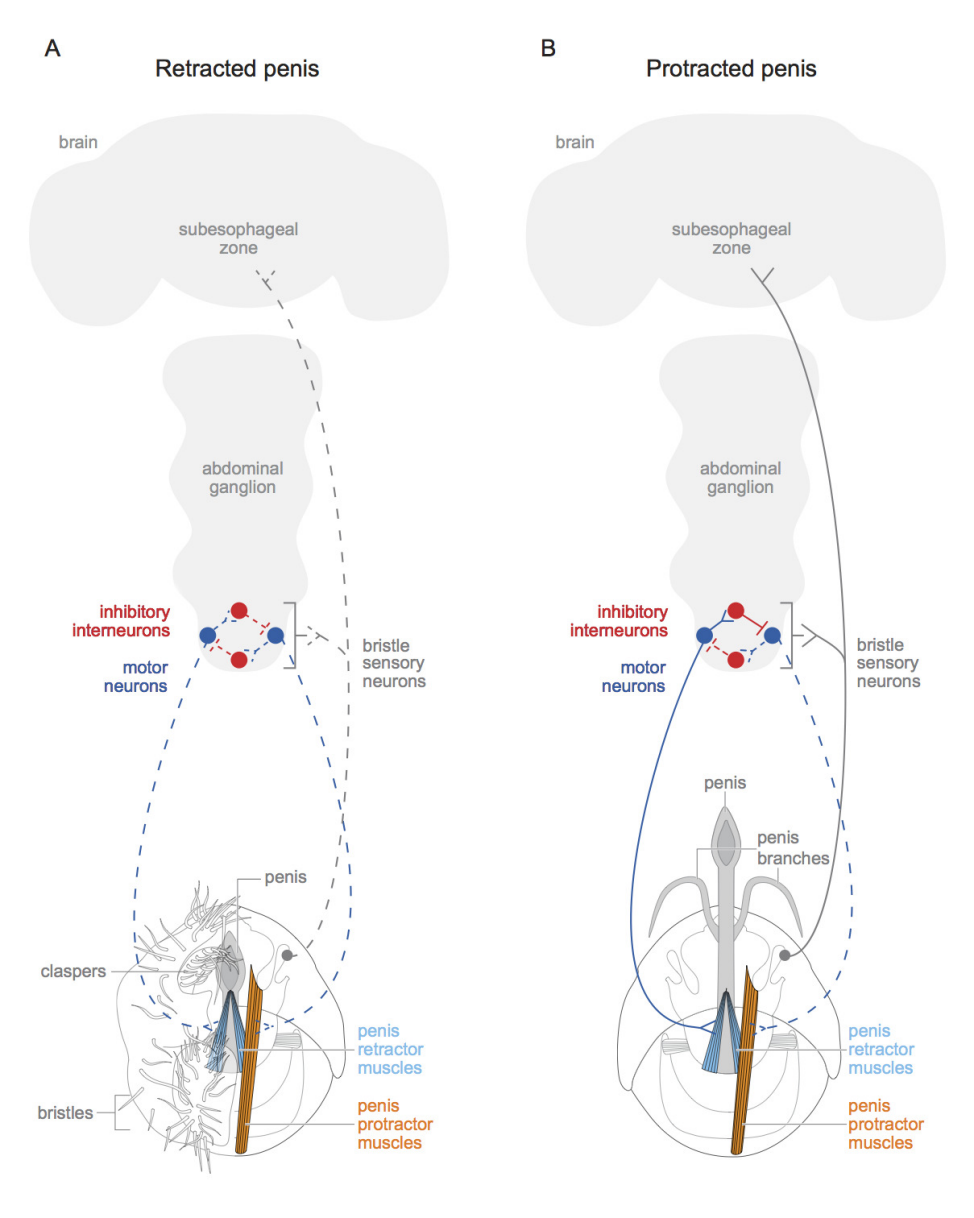
Schematic of motor circuit that controls the penis in male vinegar flies. The penis of the male vinegar fly is controlled by protractor muscles (shown in orange) and retractor muscles (light blue). (**A**) The penis will be retracted if all of the *doublesex*-positive motor neurons (blue) are inactive (indicated by dashed lines), or if neuromuscular activity if actively inhibited (not shown). The sensory neurons of the genital bristles (dashed grey) innervate the abdominal ganglion and the subesophageal zone of the brain, and also connect with both the motor neurons and the inhibitory neurons. This likely aids the male in adopting the correct posture to copulate successfully. It is also possible that stimulation of the bristles activates the sensory neurons (dashed grey line) and may have some hedonic value for the male fly. The claspers, complete with bristles, are only shown on one side. (**B**) An active *doublesex*-positive motor neuron (solid blue line) causes the protractor muscle to contract, while an inhibitory interneuron (solid red line) inhibits the motor neuron (dashed blue line) connected to the retractor muscles. This extends the penis and its branches. The muscles with no designated colour have unknown functions, and the protractor muscles are only depicted on one side. Schematics of the genitalia were kindly provided by Janice J Ting.

Finally, Pavlou et al. also report that the circuit that controls the muscles that move the penis is independent of the one that controls ejaculation. This indicates that the control of copulation is separate from that of reproduction in male vinegar flies. It also hints that copulation should be considered behaviour in its own right, and possibly one with hedonic value (in other words, one that male vinegar flies might ‘enjoy’).

A number of outstanding questions remain. For example, does an equivalent circuit control the genitalia of female flies during copulation? Moreover, how do the results of Pavlou et al. link to recent studies that explored the neural circuitry controlling the persistence and motivation of male flies during copulation (Crickmore and Vosshall, 2013; Zhang et al., 2016)? And how do male and female flies coordinate their behaviour to increase the chances of successful reproduction?
